# Updates in the Management of EGFR-Mutated Non-small Cell Lung Cancer (NSCLC)

**DOI:** 10.1007/s11912-026-01751-0

**Published:** 2026-04-11

**Authors:** Abhinav Vyas, Won Jin Jeon, Sanad Alhushki, Eric Singhi, Fawzi Abu Rous, Rajat Thawani, Aakash Desai

**Affiliations:** 1 Division of Medical Oncology and Hematology, Loma Linda University, Loma Linda, CA USA; 2 O’Neal Comprehensive Cancer Center, University of Alabama at Birmingham, Birmingham, AL USA; 3 Department of General Oncology, Division of Cancer, Medicine, MD Anderson Cancer Center, Houston, TX USA; 4 Medical Oncology, Henry Ford Health, Detroit, MI USA; 5 Division of Hematology/Medical Oncology, Oregon Health & Science University, Portland, OR USA

**Keywords:** EGFR-mutant, Non-small cell lung cancer, Targeted therapy

## Abstract

**Purpose of Review:**

With the approvals of various targeted therapies including tyrosine kinase inhibitors to antibody–drug conjugates, the treatment landscape of patients with EGFR-mutant non-small cell lung cancer has drastically improved. The use of EGFR-targeted therapies has shifted to include not only advanced/ metastatic disease but also to early-stage non-small cell lung cancer.

**Recent Findings:**

Novel therapy options have sought to address the need for effective agents against acquired resistance mechanisms in EGFR mutations. Further, the role of tyrosine kinase inhibitors against EGFR mutation in the perioperative setting has been most recently evaluated. With early outcomes, clinicians are met with the need to guide patients through various treatment regimens, and mature survival results are anticipated for several key trials. Of import, the growing focus on personalized treatment options based on outcomes, quality-of-life, toxicity, tolerability, and various disease-related factors warrants consideration.

**Summary:**

This review comprehensively describes frontline and subsequent-line therapy options for patients with early stage, unresectable/ locally advanced, and advanced/ metastatic stages of EGFR-mutant non-small cell lung cancer, including unprecedented therapeutic agents.

## Introduction

Lung cancer is currently the most frequently diagnosed cancer worldwide and the leading cause of cancer-related deaths, with roughly 2 million diagnoses and 1.8 million deaths annually. In the United States alone, around 240,000 new diagnoses and 130,000 deaths are reported each year. However, due to advancements in screening, prevention, and management options including targeted therapies, immunotherapy, and novel therapies, survival rates are steadily improving, offering hope in the fight against this devastating disease.

Lung cancer can be categorized into two main histologies: small-cell lung cancer and non-small-cell lung cancer (NSCLC) [1–3]. NSCLC is the most prevalent type of lung cancer, comprising approximately 85% of all cases. About one-third of patients with NSCLC have an activating EGFR mutation, making them potential candidates for targeted therapies, which provide a more personalized and effective treatment approach. While most studies primarily focus on advanced NSCLC, the prevalence of *EGFR* mutations varies significantly across regions. It is lowest in Europe (14.1%) and the Middle East/North Africa (17.2%) but reaches its highest levels in Asia, accounting for 49.1% of cases.

A meta-analysis of 115,815 patients with NSCLC revealed comparable *EGFR* mutation rates across stages, ranging from 29.9% to 34.0% in stages I–III and 37.5% in stage IV [4, 5]. In clinical practice, next-generation sequencing (NGS) has revolutionized the detection of common and rare EGFR mutations and other genetic alterations [6]. This advancement has paved the way for using EGFR tyrosine kinase inhibitors (TKIs), significantly improving overall survival and progression-free survival in patients with EGFR-mutant (EGFRm) NSCLC. In NSCLC, there are various EGFR activating, resistance and uncommon mutations, with specific treatment implications. EGFR exon 19 deletion and L858R point mutation in exon 21 comprise 70–85% of EGFR mutations. The most common resistance EGFR mutations include T790 point mutations insertion mutations in exon 20 [7]. There are at least three generations of EGFR-TKIs: first-generation agents (erlotinib, gefitinib), second-generation agents (afatinib, dacomitinib), and third-generation agents (Osimertinib, furmonertinib, aumolertinib, lazertinib). In addition, various antibody–drug conjugates (ADCs) and targeted therapies have emerged [8–10].

**Fig. 1:**
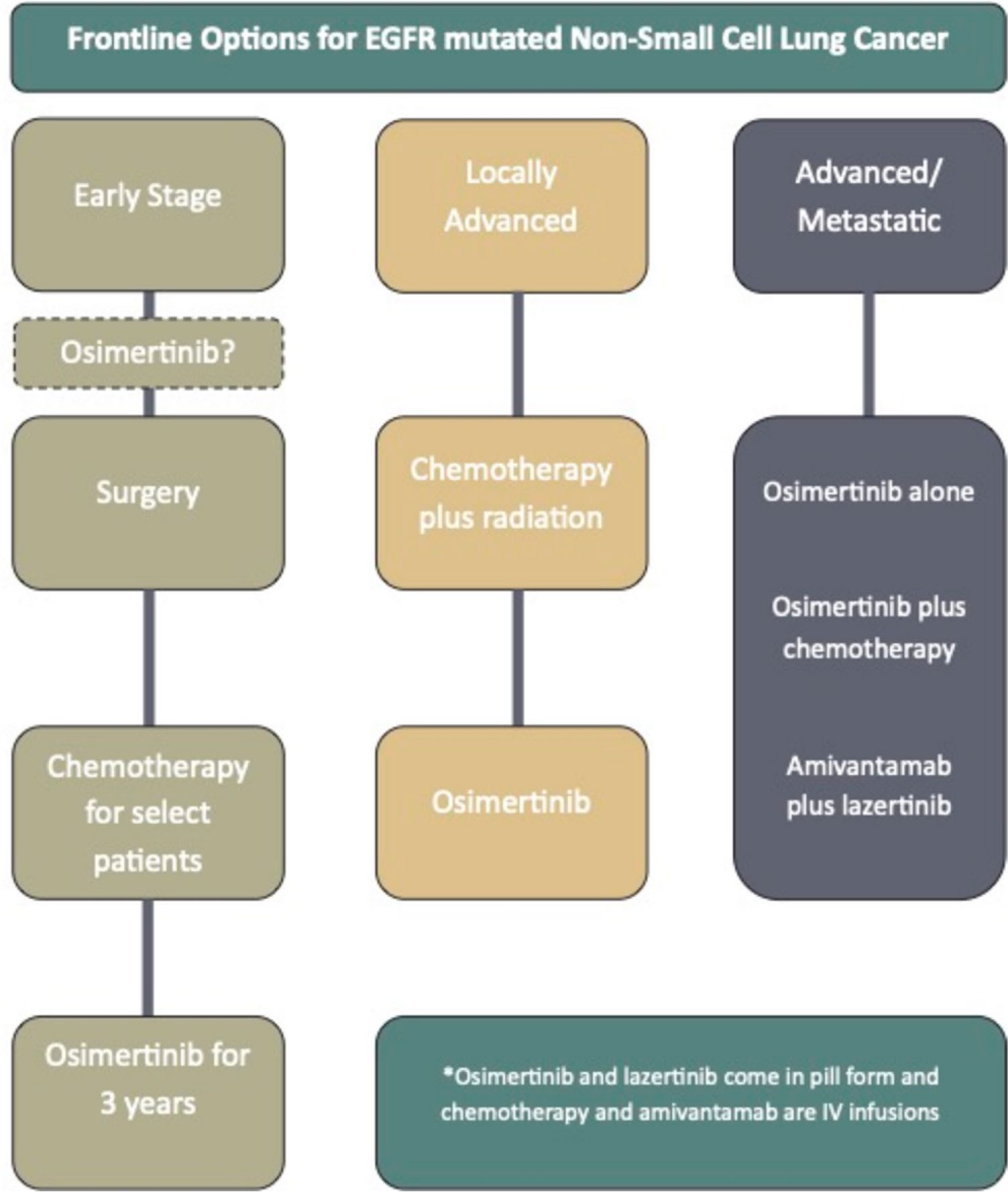
Frontline treatment options for EGFR-Mutated NSCLC**.** Overview of current first-line treatment strategies for EGFR-mutated NSCLC based on disease stage

Shared decision-making is crucial in managing metastatic EGFR-mutant non-small cell lung cancer (EGFRm NSCLC) because of the complexity of initial treatment options and the evolving evidence in this area [11]. Clinicians and patients need to weigh the proven efficacy and safety against the potential long-term advantages various approved upfront therapies, which carry toxicity and have impact on quality of life (Fig. 1).

While these therapies have yielded promising results, challenges remain. There is a pressing need for innovative strategies to enhance and predict clinical responses, overcome resistance mechanisms in EGFRm NSCLC, and improve overall survival (OS) and quality of life (QOL) for patients [12, 13]. This review highlights the latest updates in advancements and management strategies for EGFRm NSCLC, offering an in-depth exploration of progress in this evolving field.

## Advanced/ Metastatic Stage EGFRm NSCLC

The treatment landscape of EGFRm NSCLC has advanced, starting from unresectable, advanced/ metastatic setting and recently to the resectable, early stages. From single-agent to combination strategies, the first and subsequent-line therapies for EGFRm NSCLC have expanded (Table 1).

**Table 1:** Key clinical trials in EGFR-Mutated NSCLC

Trial name	Phase	Population	Intervention	Comparato	Key outcomes	Severe toxicities
FLAURA	Phase 3	EGFR-mutant advanced NSCLC (1st line)	Osimertinib	Gefitinib/ Erlotinib	Median PFS: 18.9 vs. 10.2 months, HR: 0.46 (*p* < 0.001	Grade 3- Diarrhea(3%), reduced appetite (3%), anemia (3%)
ADAURA	Phase 3	Resected stage IB-IIIA EGFR-mutant NSCLC	Osimertinib (adjuvant)	Placebo	4-year DFS: 70% vs. 29% in stage II-IIIA, HR: 0.18 (*p* < 0.001)	Grade 3- Diarrhea(2%), paronychia (2%) Grade 2- Pruritis(2%)
LAURA	Phase 3	Unresectable stage III EGFR-mutant NSCLC	Osimertinib (consolidation)	Placebo	Median PFS: 39.1 vs. 5.6 months, HR: 0.23 (*p* < 0.001)	Grade 3- Radiation pneumonitis (10.49%), pneumonia(4.9%),
FLAURA2	Phase 3	EGFR-mutant advanced NSCLC (1st line)	Osimertinib + chemotherapy	Osimertinib	Median PFS: 25.5 vs. 16.9 months, HR: 0.62	Grade 4-Neutropenia(3%), anemia(20%), diarrhea(3%),
NEOADAURA	Phase 2	Resectable EGFR-mutant stage II-IIIB NSCLC	Osimertinib (neoadjuvant)	-	Major pathological response in early results	Grade 3- neutropenia(11%), thrombocytopenia(8%)
MARIPOSA	Phase 3	EGFR-mutant advanced NSCLC (1st line)	Amivantamab + Lazertinib	Osimertinib	Ongoing	Grade 3- Pneumonia(4%), PE(6.18%), Pleural effusion (2%)
MARIPOSA-2	Phase 3	EGFR-mutant advanced NSCLC (post-TKI)	Amivantamab-based combinations	Chemotherap y	Improved PFS with amivantamab combinations	Grade 3- Neutropenia(55%), Thrombocytopenia(37%), anemia(18%)
CHRYSALIS	Phase 1	EGFR-mutant advanced NSCLC (post-TKI)	Amivantamab + Lazertinib	-	Promising antitumor activity in osimertinib-relapsed NSCLC	Grade 3- Dyspnea(4%), Rash(3%), Hypokalemia(5%),
ASCENT	Phase 2	Locally progressive EGFR-mutant NSCLC	Afatinib (induction)	-	Significant ORR and MPR with manageable toxicity	Grade 4- Rash(5%), diarrhea(5%), esophagitis(3%)
NEOS	Phase 2	Resectable EGFR-mutant stage II-IIIB NSCLC	Osimertinib (neoadjuvant)	-	ORR: 71.1%, R0 resection: 93.8%, manageable adverse events	Grade 3- Rash(3%) Grade 2- oral ulcers(3%), fatigue(3%)
RAMOSE	Phase 3	EGFR-mutant advanced NSCLC	Osimertinib + Ramucirumab	Osimertinib	Improved PFS with manageable toxicity	Grade 4- Hyponatremia(1.1%) Grade3- Hypertension(22.6%), diarrhea(3.2%)
HERTHENA-Lung	Phase 2	EGFR-mutant NSCLC (post-TKI, post-PBC)	HER3-DXd	-	ORR: 29.8%, median OS: 11.9 months, CNS ORR: 33.3%	Grade > 3- Thrombocytopneia(21%), neutropenia(19%), anemia(14%)

Summary of pivotal clinical trials investigating targeted therapies in EGFR-mutated NSCLC

Osimertinib demonstrated superior efficacy compared to gefitinib or erlotinib in the frontline treatment of metastatic EGFR-mutant NSCLC, as shown in the phase 3 FLAURA trial, leading to its wide use as monotherapy for treatment naïve patients with unresectable, advanced EGFRm NSCLC [14, 15]. The FLAURA trial, a landmark phase 3 study, demonstrated the superior efficacy of osimertinib, a third-generation EGFR-TKI that targets mutations in EGFR exon 19 deletion, exon 21 L858R, and T790M, in the first-line treatment of previously untreated advanced EGFRm NSCLC [11]. Osimertinib 80 mg daily significantly improved both progression-free survival (PFS) compared to first-generation EGFR-TKIs (gefitinib or erlotinib) with median PFS 18.9 months vs 10.2 months, HR 0.46, 95% CI 0.37–0.57, *p* < 0.001) with superior median duration of response and OS (median OS 38.6 months vs 31.8 months, HR 0.80, 95% CI 0.64–1.00, *p* = 0.046). Additionally, the drug proved effective in patients with the T790M resistance mutation, which commonly emerges during earlier EGFR-TKI treatments [16, 17].

Osimertinib also showed strong efficacy in addressing CNS metastases, a frequent and challenging complication in NSCLC, further establishing its value as a first-line treatment for advanced EGFRm NSCLC [17, 18]. The most recent update of FLAURA demonstrated improved median OS of 38.6 months, 95% CI 34.5–41.8 vs 31.8 months, 95% CI 26.6–36.0 months, HR 0.80, 95% CI 0.64–1.00, *p* = 0.046) [19]. As such, osimertinib monotherapy remains a highly effective and well-tolerated regimen for treating advanced NSCLC with *EGFR* mutations. Treatment goals should focus not only on survival but also on improving quality of life. While single agent osimertinib is suitable for many patients, the rise of combination therapies marks a new era of personalized care and broader treatment possibilities [20].

Osimertinib has established itself as an effective first-line therapy for advanced NSCLC with EGFR mutations [11]. However, disease progression and the emergence of resistance remain significant challenges. Recent research suggests that combining osimertinib with additional treatments may help delay resistance and improve outcomes. Building on this efficacy profile of osimertinib, the FLAURA2 trial demonstrated improved PFS and OS with osimertinib in combination with platinum-based chemotherapy in nonsquamous advanced/ metastatic EGFRm NSCLC. The FLAURA2 trial, a phase 3 study, demonstrated that first-line treatment with osimertinib plus chemotherapy (pemetrexed and a platinum-based agent) significantly improves PFS compared to osimertinib monotherapy (median PFS 25.5 months vs 16.7 months, HR 0.62, 95% CI, 0.49–0.79, *p* < 0.001) as well as OS (median OS 47.5 months vs 37.6 months, HR 0.77, 95% CI, 0.61–0.96, *p* = 0.02) [21, 22]. This prolonged therapeutic effect was further supported by a longer median duration of response, aligning with findings from trials of first-generation EGFR-TKIs combined with chemotherapy [21]. However, the combination of osimertinib and platinum-pemetrexed was associated with a higher incidence of grade 3 adverse events, primarily hematologic toxicities consistent with chemotherapy-induced bone marrow suppression. Gastrointestinal side effects were also more frequent in the combination group. Despite these toxicities, dose interruptions had minimal impact on osimertinib exposure, suggesting that the treatment remains manageable.

Additionally, the recent phase II RAMOSE trial concluded that adding ramucirumab to osimertinib improved PFS in EGFRm treatment naive, metastatic NSCLC patients compared to osimertinib monotherapy [22]. The interim analysis showed that adding ramucirumab to osimertinib significantly increased median PFS compared to osimertinib alone (24.8 months vs 15.6 months, HR 0.55, 95% CI 0.32 to 0.93; *P* = 0.026). However, there were no significant differences in the overall response rates (ORRs). Still, combination therapy with ramucirumab plus osimertinib was also safe and well-tolerated. As this has not yet been approved for use, further analysis and updates are awaited.

Besides osimertinib, there have been other third-generation TKIs that have shaped the landscape of advanced/ metastatic EGFRm NSCLC. In the LASER301 study, lazertinib as a was evaluated as first-line treatment for EGFRm advanced NSCLC [15]. The study demonstrated that lazertinib provides significantly PFS compared to gefitinib, with consistent efficacy across various subgroups. Its manageable adverse event profile also sets it apart from other TKIs, further establishing its potential as a promising treatment option. However, the use of lazertinib was approved not as monotherapy but in combination with amivantamab, a bispecific antibody against EGFR and MET receptors based on the results from the MARIPOSA trial. The MARIPOSA trial, a phase III study involving patients with treatment-naïve advanced EGFRm NSCLC compared the efficacy and safety of amivantamab plus lazertinib against osimertinib monotherapy. Combination therapy with amivantamab plus lazertinib significantly reduced the risk of disease progression or death by 30% (median PFS 23.7 months vs 16.6 months, HR 0.70, 95% CI 0.58–0.85, *p* < 0.001). Secondary endpoints, such as duration of response (DOR), also indicated notable improvements with the combination therapy. After median follow up of 37.8 months, the MARIPOSA trial demonstrated significantly longer median OS for the amivantamab + lazertinib combination (not reached (42.9 months – not reached)) compared to median OS of 36.7 months (33.4–41.0 months) for the osimertinib monotherapy arm (HR 0.75, 95% CI 0.61–0.92, *p* < 0.005) [23]. Common treatment-related adverse events included paronychia, rash, and venous thromboembolism, with grade 3 or higher events occurring more frequently in the combination arm compared to osimertinib monotherapy (75% vs. 43%) [24]. At this time, the National Comprehensive Cancer Network (NCCN) and European Society for Medical Oncology (ESMO) guidelines recommend osimertinib monotherapy, combination osimertinib plus chemotherapy, and amivantamab plus lazertinib for first-line therapy in advanced / metastatic EGFRm NSCLC.

In addition to osimertinib and lazertinib, other third-generation TKIs, such as alflutinib, rezivertinib, and abivertinib, have been approved in different regions, further enriching the treatment landscape for EGFR-mutated NSCLC. Alflutinib (AST2818 or furmonertinib) has shown impressive results in managing EGFR T790M-positive NSCLC, with high objective response rates (ORRs) and favorable PFS in clinical trials, leading to its approval in China. Almonertinib, another third-generation EGFR-TKI, has been approved by the Chinese National Medical Products Administration for patients with EGFR-mutated NSCLC who have progressed on prior EGFR-TKI therapy. It showed an impressive response rate, disease control rate, and progression-free survival, with particularly notable efficacy in patients with CNS metastases. Almonertinib marks a significant advancement in EGFR-targeted therapies in China and serves as a valuable complement to osimertinib, which is already established as a frontline treatment for EGFR-mutated NSCLC [25].

Several novel EGFR-TKIs are under development. Rezivertinib (BPI-7711) has demonstrated potent inhibition of EGFR mutations and excellent CNS penetration in preclinical studies [26]. Clinical trials further support its efficacy, showing significant ORRs and PFS benefits, though myelosuppression remains a notable side effect. Abivertinib (AC0010/Avitinib/STI-6565) has also emerged as a promising agent, effectively targeting *EGFR* T790M mutations with encouraging clinical trial results [27, 28]. While its recommended phase 2 dose (RP2D) has shown manageable side effects, ongoing randomized trials comparing it to gefitinib aims to further clarify its therapeutic potential. Together, these agents reinforce the expanding landscape of third-generation EGFR TKIs, offering new hope for patients with advanced EGFRm NSCLC, particularly those with T790M resistance or CNS involvement.

In this context, a randomized phase 2 trial explored the combination of osimertinib with carboplatin-pemetrexed in NSCLC patients with EGFR mutations who had progressed after initial EGFR-TKI therapy [29]. While the combination therapy did not significantly improve progression-free survival (PFS) compared to osimertinib alone, it was deemed safe and manageable despite a slightly higher incidence of non-hematologic adverse events. This study represents an important step in investigating combination therapies for the first-line management of EGFRm NSCLC, highlighting the need for continued innovation in treatment strategies.

## Resistance Mechanisms and Implications for Second Line Therapy

Identifying activating mutations in the EGFR gene has revolutionized the treatment of NSCLC. Targeted therapies using EGFR-TKIs have significantly improved outcomes [30, 31]. However, the emergence of resistance—most commonly due to the T790M mutation in *EGFR* exon 20—has posed a significant challenge. Third-generation TKIs like osimertinib were developed to address this, targeting both activating *EGFR* mutations and the T790M resistance mutation. Clinical trials have shown osimertinib to be highly effective in prolonging PFS and achieving high ORRs, both as first line treatment and in salvage therapy [3]. Importantly, osimertinib has also demonstrated efficacy in managing central nervous system (CNS) metastases, a frequent complication in NSCLC.

Despite its initial success, resistance to osimertinib inevitably develops, limiting its long-term efficacy. Resistance mechanisms can be categorized as EGFR-dependent or EGFR-independent. EGFR-dependent resistance involves acquired mutations such as C797S, G796R, L792, L718, and G724, which hinder osimertinib’s ability to bind to EGFR, leading to varying resistance [1]. These mutations may occur alone or in combination, complicating treatment strategies. On the other hand, EGFR-independent resistance mechanisms involve alternative bypass pathways, such as MET and HER2 amplifications, aberrant downstream signaling through the RAS-MAPK and PI3K pathways, and histologic transformations, including small-cell lung cancer (SCLC) [2, 32, 33]. Additionally, epithelial-to-mesenchymal transition (EMT) and alterations in cell-cycle genes have been associated with osimertinib resistance.

Addressing these resistance mechanisms is critical for improving patient outcomes. Fourth-generation allosteric EGFR-TKIs, which are orally bioavailable and selectively target EGFR-activating mutations, offer a promising approach. These agents can overcome C797S-mediated resistance by binding to an allosteric site on EGFR, altering its conformation, and bypassing resistance. While preclinical studies have shown potent efficacy, only a few of these agents have advanced to clinical trials, underscoring the need for further drug development. Combination therapies and novel agents targeting specific mutations are also under investigation to combat resistance more effectively [34].

Research efforts remain focused on understanding and overcoming resistance, with ongoing tumor profiling and patient monitoring playing a key role in adapting treatment strategies. In addition, the potential role of circulating tumor DNA (ctDNA) and liquid biopsy along with the ability to detect resistance mutations while on EGFR-TKIs is an area of relevance. There are ongoing prospective trials evaluating the use of biomarkers including ctDNA and minimal residual disease (MRD) in EGFRm NSCLC [35]. The detection of EGFR-dependent and independent resistance mutations at progression of disease or lack of clinical response via ctDNA could aid clinicians in navigating various targetable mutations. Despite the challenges, osimertinib remains a cornerstone in treating EGFRm NSCLC, offering significant benefits in first line and salvage settings, particularly in managing CNS metastases. The evolving landscape of EGFR-targeted therapies holds promise for further improving outcomes in this patient population.

Uncommon EGFR mutations, excluding exon 20 insertions, have historically lacked prospective clinical evidence, leaving their optimal management uncertain. Afatinib is one of the few FDA-approved therapies in the United States, supported by a post hoc pooled analysis of the LUX-Lung 2, 3, and 6 trials, which showed ORR of 71.5% and a median PFS of 11 months [36]. However, over half of the patients required dose reductions due to dermatologic and gastrointestinal toxicities.

Recent prospective data evaluating osimertinib have expanded treatment options for patients with uncommon EGFR mutations. The phase II UNICORN trial enrolled 42 treatment-naïve Japanese patients with mutations such as G719X, S768I, E709X, or compound uncommon mutations and demonstrated an ORR of 55%, a median PFS of 9.4 months, and a notably long median duration of response (DOR) of 22.7 months [B]. Improved responses were observed in patients with L861Q and compound mutations, and the treatment was generally associated with manageable toxicity. Collectively, these findings—emphasized by the prospective UNICORN trial—establish osimertinib as a viable first-line therapy for advanced NSCLC with uncommon EGFR mutations [37, 38]. Afatinib also remains a reasonable option, with earlier pooled analyses suggesting comparable response rates but somewhat longer progression-free survival, although these analyses predate contemporary osimertinib trials.

Potential therapeutic strategies to overcome resistance, including combination therapies targeting specific pathways and inhibitors of key molecules involved in resistance mechanisms. Preclinical and clinical evidence supporting these strategies is discussed, emphasizing the need for personalized approaches to treatment based on the underlying resistance mechanisms identified in individual patients.

## Systemic Progression, Subsequent-line and Beyond

In response to resistance mechanisms in EGFRm NSCLC, various therapy options for subsequent lines after systemic progression on EGFR-targeted therapies have been evaluated and approved. MARIPOSA-2, a phase 3 study, led to the approval of amivantamab plus chemotherapy for use in EGFRm NSCLC after progression on osimertinib. When combined with chemotherapy, amivantamab with or without lazertinib demonstrated improved PFS compared to chemotherapy alone (median PFS 6.3 months vs 8.3 months vs 4.2 months, HR 0.41 and 0.38, *p* < 0.001, respectively). The study highlights the importance of targeting different resistance mechanisms, such as EGFR and MET alterations, which amivantamab has shown efficacy against, in combination with chemotherapy's activity against other resistance pathways. Amivantamab-based treatments have shown promising results in improving intracranial progression-free survival, even without a CNS-active agent like lazertinib. However, there are concerns about hematologic adverse events, particularly neutropenia, in the triple therapy of amivantamab, lazertinib and chemotherapy regimen [39]. In the second interim analysis, the amivantamab plus chemotherapy combination did not meet its prespecified significance threshold for OS (median OS 17.7 months vs 15.3 months, HR 0.73, 95% CI 0.54–0.99, *p* = 0.039). Still, the final analysis from ongoing follow up of this regimen in the second-line setting is awaited [40].

The recent failures of immunotherapy-chemotherapy regimens in post-osimertinib setting highlights the efficacy demonstrated by amivantamab-based combinations, potentially setting a new standard of care for this patient population [34]. Furthermore, the MARIPOSA-2 trial's innovative approach utilizing circulating tumor DNA (ctDNA) for biomarker evaluation streamlines patient assessment without the need for pretreatment tissue biopsies, emphasizing the promising role of amivantamab-based therapies in reshaping the treatment landscape for EGFRm advanced NSCLC after progression on osimertinib.

Besides osimertinib and lazertinib, sunvozertinib, a novel EGFR-TKI, received accelerated approval based on the WU-KONG1B trial [41]. This study evaluated the role of sunvozertinib for patients with locally advanced or metastatic NSCLC with EGFR exon 20 insertions as subsequent line after platinum-based chemotherapy and including immunotherapy and amivantamab. The ORR for patients on sunvozertinib monotherapy was 46%, 95% CI 35–57%. The duration of response was about 11.1 months, 95% CI 8.2- not evaluable. The most common grade 3 or higher AEs were elevated creatine phosphokinase (CPK), diarrhea and anemia. The addition of sunvozertinib to the armamentarium of treatment options for patients who progress on previous EGFR-TKI and are heavily treated demonstrates the need for further evaluation of EGFR-targeted therapies, especially in the setting of resistance mutations.

Beyond EGFR-TKIs, recently, datopotamab deruxtecan (Dato-DXd), trophoblast cell surface antigen-2 (TROP2) directed antibody drug conjugate (ADC) with a topoisomerase I inhibitor payload, received accelerated approved for EGFRm NSCLC who progressed after EGFR-directed therapy and platinum-based chemotherapy based on the results of the TROPION-Lung05 study [42]. The trial involved 137 patients, of which 56.9% had EGFR mutations; the study showed ORR of 43.6% in those with *EGFR* mutations. The most common grade ≥ 3 treatment-emergent AEs included stomatitis (9.5%), anemia (5.8%), and increased amylase (5.8%). The safety profile was manageable and aligned with previous observations for Dato-DXd. These findings highlight Dato-DXd’s promising antitumor activity and tolerable safety in heavily pretreated NSCLC patients as subsequent line therapy after EGFR-directed therapies [43].

Another phase 2 trial evaluated the efficacy and safety of patritumab deruxtecan (HER3-DXd), an ADC of human monoclonal antibody to HER3, a member of the ERBB family of which EGFR is a part, to a topoisomerase I inhibitor payload, in patients with EGFRm NSCLC who had previously received 3rd generation EGFR-TKI [44]. While OS data has yet to mature, HER3-DXd demonstrated clinically meaningful efficacy, with improved PFS compared to platinum-based chemotherapy (median PFS 5.8 months vs 5.4 months, HR 0.77, 95% CI 0.63–0.94, *p* = 0.011) with ORR of 35.2%. The confirmed CNS ORR of 33.3% in patients with CNS metastases is particularly significant. HER3-DXd has shown promising results as a treatment option for EGFR-mutated NSCLC patients after progression on EGFR TKI therapy and PBC. It demonstrated a manageable safety profile with thrombocytopenia being the most common treatment-emergent AE. HER3-DXd has shown diverse efficacy across various subgroups, including tumors with different mechanisms of EGFR TKI resistance [39, 44, 45].

Lastly, the HARMONi-A (AK112-301) study marks a significant milestone as the first phase 3 trial demonstrating the clinical benefit of ivonescimab plus chemotherapy in patients with NSCLC who progressed on EGFR-TKIs [46]. Ivonescimab, combined with chemotherapy, significantly prolonged PFS compared to chemotherapy alone (HR, 0.46; *P* < 0.001), with consistent benefits observed across subgroups, including those with exon 19 deletions, L858R, and T790M variants. Unlike other therapies, ivonescimab’s dual targeting of VEGF and PD-1 may contribute to its unique efficacy, particularly in patients with brain metastases. This reinforces its potential as a promising option for patients resistant to third-generation TKIs, challenging the current standard of platinum-based chemotherapy alone [47].

## Early Stage EGFRm NSCLC

### Neoadjuvant Setting

Beyond the advanced, metastatic setting, the role of EGFR-directed therapies has been evaluated in the early stage, resectable setting as well. Locally advanced NSCLC, accounting for roughly 30% of cases, poses unique challenges in treatment due to its heterogeneous nature [11, 48, 49]. Among these, approximately 34% harbor *EGFR* mutations, significantly influencing treatment outcomes. Emerging evidence highlights distinct patterns of disease control in EGFRm NSCLC following platinum-based chemoradiation therapy (CRT), particularly concerning the CNS metastases [11, 50–52].

Early investigations into EGFR-TKI–based neoadjuvant therapy primarily focused on first- and second-generation inhibitors, such as erlotinib and gefitinib, which showed promising results in several single-arm phase II trials [13, 53, 54]. These studies demonstrated notable objective response rates and major pathological response (MPR) rates, with improved outcomes compared to chemotherapy. However, despite these encouraging findings, challenges remain particularly the low rates of pathological downstaging and complete response, highlighting the need for further advancements in neoadjuvant strategies [55].

Several studies have evaluated the role of EGFR-TKIs in the neoadjuvant setting. For example, a phase II clinical trial by Zhong et al. compared erlotinib to gemcitabine plus cisplatin (GC) chemotherapy in treating patients. While there was no significant difference in the overall response rate (*p* = 0.09), erlotinib showed a trend toward better results (54.1% ORR vs. 34.3% for GC). Additionally, PFS was significantly longer with erlotinib (21.5 months vs. 11.4 months for GC, *p* = 0.003). However, the study did not improve OS, as the data were still inconclusive [13].

The ASCENT study investigated the use of afatinib, a second-generation EGFR-TKI, and concurrent chemoradiation with and without surgery patients with locally advanced NSCLC [56]. This study found that induction therapy with afatinib achieved significant ORR and MPR rates with manageable toxicity. Despite these benefits, disease recurrence remains a significant concern for patients treated with surgery or radical chemoradiotherapy [57].

The NEOS trial investigated the safety and efficacy of osimertinib as neoadjuvant therapy in patients with EGFR-mutant resectable stage IIA-IIIB lung adenocarcinoma [58]. Conducted in mainland China, the trial enrolled 40 eligible patients and reported an impressive objective response rate of 71.1% among those completing the 6-week treatment course. Furthermore, 93.8% of patients achieved successful R0 resection post-treatment. Treatment-related adverse events, primarily rash, diarrhea, and oral ulceration, were generally manageable, with only a small proportion experiencing grade 3 events. These results highlight potential of osimertinib as neoadjuvant therapy for EGFRm NSCLC, showcasing both strong efficacy and an acceptable safety profile.

Similarly, the analysis of another phase II study evaluated the role of neoadjuvant osimertinib monotherapy in 27 patients with surgically resectable stage I-IIIA EGFRm NSCLC; however, it did not meet its MPR, which was 14.8%, 95% CI 4.2–33.7%. There were no complete responses observed [57].

Following this study, the recent NeoADAURA demonstrated improved outcomes with the use of neoadjuvant osimertinib with or without platinum-based chemotherapy in patients with stage II-IIIB EGFRm NSCLC [59]. Patients were assigned to osimertinib plus chemotherapy, osimertinib monotherapy, and chemotherapy alone. There was significantly improved MPR with osimertinib plus chemotherapy and osimertinib monotherapy compared to chemotherapy alone and at 15% data maturity, the HR for event-free survival (EFS) did not meet the prespecified significance (HR 0.50, 99.8% CI 0.17–1.41, *p* = 0.04). Though pending data maturation, the use of neoadjuvant osimertinb in the neoadjuvant setting is promising.

### Adjuvant Setting

Though the role of EGFR-TKIs such as osimertinib in the neoadjuvant setting is being explored, the use of adjuvant osimertinib is standard practice at this time. The potential role of EGFR-TKIs as adjuvant therapy in NSCLC patients has long been a subject of debate [60]. Over the years, multiple studies have reported conflicting outcomes regarding the effectiveness of adjuvant EGFR-TKIs in resected NSCLC. For instance, trials such as IMPACT, RADIANT, BR19, and the work by Feng et al. consistently indicated that adjuvant treatments with erlotinib, gefitinib, or icotinib did not yield significant survival benefits for patients with resected NSCLC [61–63].

However, osimertinib has become a widely recommended adjuvant treatment for patients with resected stage IB to IIIA EGFRm NSCLC [64, 65]. Its approval was based on the results of the landmark ADAURA trial, a phase III, double-blinded, randomized study that evaluated the efficacy of osimertinib compared to placebo in patients with completely resected stage IB to IIIA EGFRm NSCLC with or without adjuvant chemotherapy. The trial demonstrated that osimertinib significantly improved DFS and provided substantial OS benefits [66]. Among patients with stage II to IIIA disease, the 4-year DFS rate was 70% for osimertinib versus 29% for placebo, while the 5-year OS rate was 85% for osimertinib and 73% for placebo (HR 0.49, 95% CI 0.33–0.73, *p* < 0.001). These benefits were observed consistently across all predefined subgroups [67, 68]. Moreover, osimertinib markedly reduced metastatic disease, particularly in the CNS. The CNS DFS hazard ratio was 0.18 (95% CI, 0.10 to 0.33) [69]. CNS metastases, a frequent and devastating complication in NSCLC, are associated with poor prognosis and diminished quality of life [19]. In the CNS DFS analysis for stage II to IIIA disease, only 3 of 18 patients in the osimertinib group experienced CNS recurrence as their first site while on treatment, compared to 29 of 32 patients in the placebo group.

Furthermore, efficacy benefit from the trial may indeed have been underestimated, owing to the fact that a protocol modification allowed eligible patients to receive open label osimertinib at recurrence, which was implemented after the primary analysis. Despite this, the 5-year OS benefit was maintained, highlighting the significant role of osimertinib in the adjuvant setting for EGFRm NSCLC [66, 70–73].

### Post-Chemoradiation for Unresectable Disease

Earlier studies, such as the phase II RTOG1306 trial, explored erlotinib in combination with CRT and suggested improved progression-free survival with an acceptable safety profile, although the trial was discontinued early [74].

More recently, the LAURA trial demonstrated that osimertinib significantly improves survival outcomes in patients with unresectable stage III EGFR-mutant NSCLC following chemoradiotherapy [48, 75]. The study initially demonstrated improved PFS with osimertinib (39.1 months vs 5.6 months, HR 0.16, 95% CI 0.10–0.24, *p* < 0.001). At interim analysis at 20% data maturity, OS showed a trend in favor of osimertinib (HR 0.81, 95% CI 0.42–1.56, *p* = 0.530), with 81% of patients in the placebo arm receiving osimertinib after progression. Then, at 31% maturity, there was continued trend towards improved OS with osimertinib compared to placebo.

## Conclusion

**Fig. 2:**
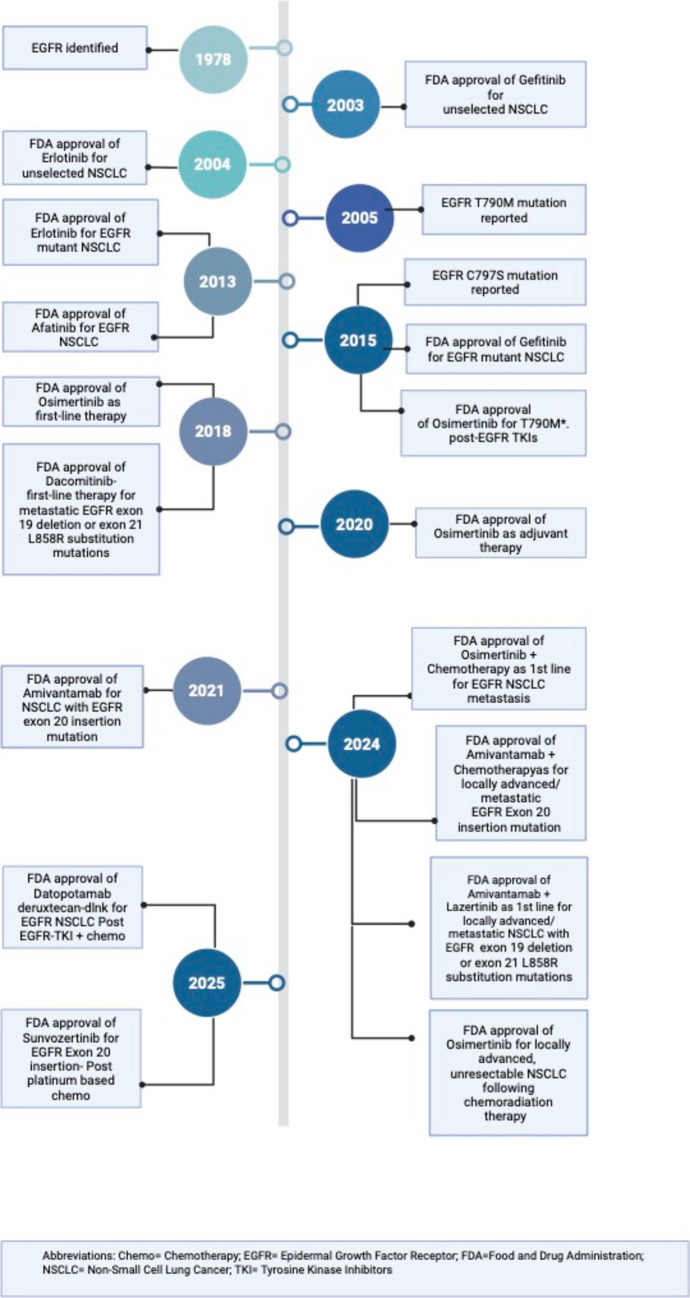
Timeline of FDA Approvals for EGFR-Mutated NSCLC Therapies**.** Timeline showing major milestones in the approval of therapies for EGFR-mutated NSCLC, from EGFR discovery to the latest first-line and post-resistance combination regimens

**Fig. 3:**
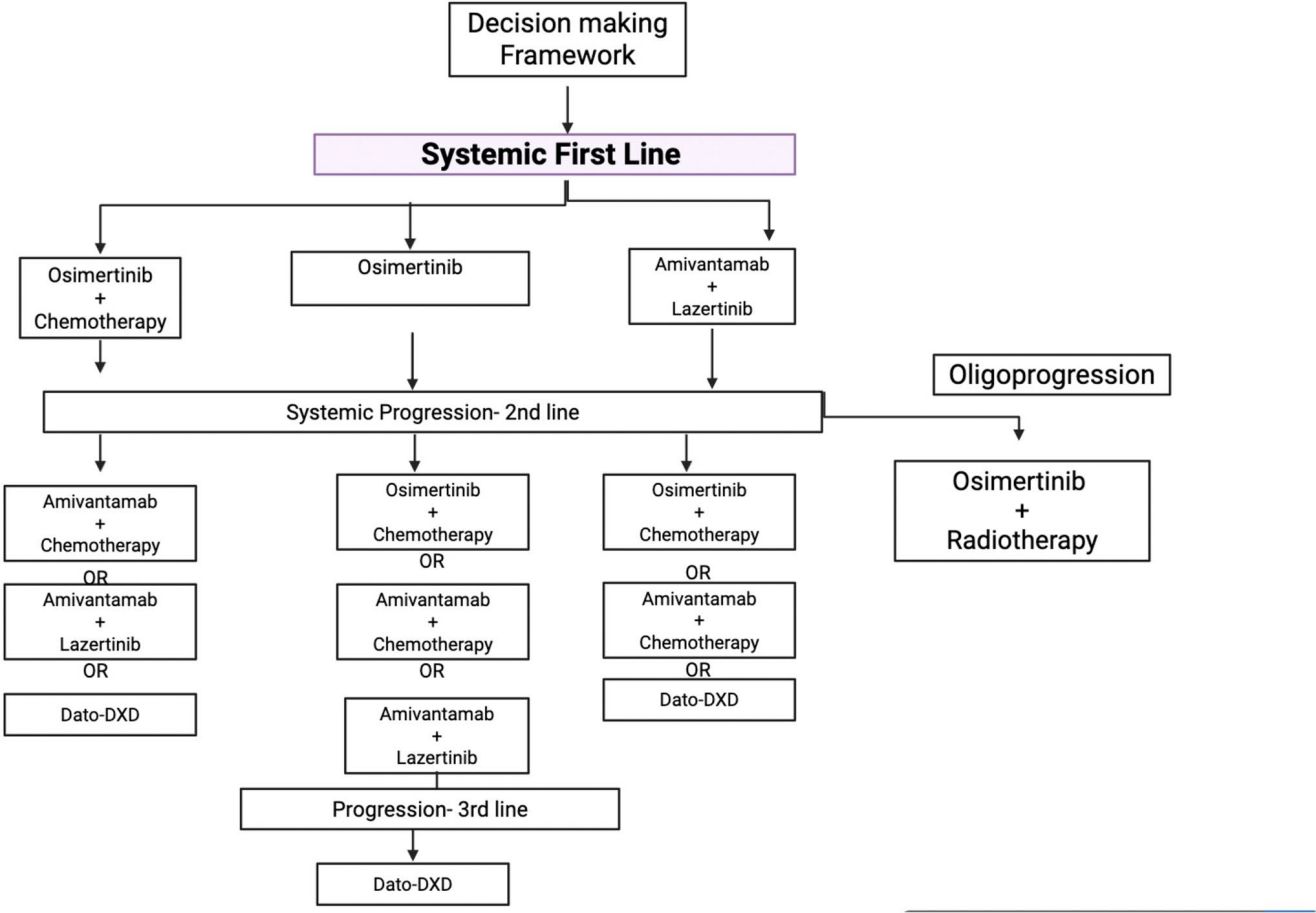
Decision making framework while chosing systemic therapy for EGFR Mutated NSCLC

The treatment of EGFRm NSCLC has undergone significant advancements in recent years, with targeted therapies including TKIs and ADCs improving outcomes for patients in various treatment settings (Fig. 2). EGFR mutations play a crucial role in NSCLC pathogenesis and are present in a significant subset of patients. EGFR-TKIs have revolutionized treatment paradigms, offering better outcomes and prolonged survival for patients. The progression of EGFR-targeted therapy from first-generation to third-generation TKIs, such as osimertinib, has further refined treatment approaches, particularly in the context of early-stage and advanced NSCLC. Specifically, for osimertinib, recent trials have emphasized the efficacy of osimertinib in the neoadjuvant, adjuvant, and consolidation settings, demonstrating significant >improvements (Fig. 3).

Alternative third-generation EGFR TKIs have the potential to offer more affordable treatment options, benefiting patients while addressing pricing monopolies [76]. Clinical trials suggest that their efficacy is comparable to osimertinib, though carefully considering their differing side effect profiles is essential when choosing the most suitable therapy [77–79]. Meanwhile, fourth-generation EGFR TKIs are under development to overcome resistance challenges, but further research is needed to define their efficacy and role in treatment. Continued investigation and clinical trials remain vital to fully evaluate the potential of these emerging therapies in improving outcomes for patients with EGFR-mutated NSCLC [17, 77, 80].

Furthermore, ongoing research is exploring novel therapeutic avenues and addressing resistance mechanisms, particularly in patients who develop resistance to first-line EGFR TKIs like Osimertinib (Fig. 2). Strategies such as combination therapies and the development of fourth-generation EGFR TKIs and novel therapeutics show promise in overcoming resistance and improving patient outcomes. The evolving landscape of EGFRm NSCLC management emphasizes the importance of personalized treatment approaches and ongoing clinical investigation. With continued advancements in targeted therapies and a deeper understanding of resistance mechanisms, the outlook for patients with EGFRm NSCLC is increasingly optimistic, offering hope for improved survival and quality of life.

## Key References

- Soria JC, Ohe Y, Vansteenkiste J, et al. Osimertinib in Untreated EGFR-Mutated Advanced Non-Small-Cell Lung Cancer. *N Engl J Med*. 2018;378(2):113-125.
  - ○ This trial introduced osimertinib as a superior EGFR-TKI for advanced, metastatic non-small cell lung cancer.
- Blakely CM, Urisman A, Gubens MA, et al. Neoadjuvant Osimertinib for the Treatment of Stage I-IIIA Epidermal Growth Factor Receptor-Mutated Non-Small Cell Lung Cancer: A Phase II Multicenter Study. *J Clin Oncol.* 2024;42(26):3105-3114.
  - ○ This trial extended to role of osimertinib to the neoadjuvant setting of early stage non-small cell lung cancer, shifting the paradigm of neoadjuvant therapy options for EGFR-mutant non-small cell lung cancer.
- Herbst RS, Wu YL, John T, et al. Adjuvant Osimertinib for Resected EGFR-Mutated Stage IB-IIIA Non-Small-Cell Lung Cancer: Updated Results From the Phase III Randomized ADAURA Trial. *J Clin Oncol.* 2023;41(10):1830-1840.
  - ○ This trial highlighted the role of osimertinib in the adjuvant setting in the early stage non-small cell lung cancer
- Planchard D, Janne PA, Cheng Y, et al. Osimertinib with or without Chemotherapy in EGFR-Mutated Advanced NSCLC. *N Engl J Med*. 2023;389(21):1935-1948.
  - ○ This trial established osimertinib plus chemotherapy as one of the first front-line options in advanced, metastatic EGFR-mutant non-smell cell lung cancer
- Cho BC, Felip E, Hayashi H, et al. MARIPOSA: phase 3 study of first-line amivantamab + lazertinib versus osimertinib in EGFR-mutant non-small-cell lung cancer. *Future Oncol.* 2022;18(6):639-647.
  - ○ This trial introduced the combination of amivantamab plus lazertinib as a front-line option alongside osimertinib monotherapy and osimertinib plus chemotherapy in EGFR-mutant non-small cell lung cancer
- Yu HA, Goto Y, Hayashi H, et al. HERTHENA-Lung01, a Phase II Trial of Patritumab Deruxtecan (HER3-DXd) in Epidermal Growth Factor Receptor-Mutated Non-Small-Cell Lung Cancer After Epidermal Growth Factor Receptor Tyrosine Kinase Inhibitor Therapy and Platinum-Based Chemotherapy*. J Clin Oncol.* 2023;41(35):5363-5375.
  - ○ This is one of the investigational trials evaluating the role of ADCs against EGFR-mutant non-small cell lung cancer
- Paz-Ares L, Ahn MJ, Lisberg AE, et al. 1314MO TROPION-Lung05: Datopotamab deruxtecan (Dato-DXd) in previously treated non-small cell lung cancer (NSCLC) with actionable genomic alterations (AGAs). *Annals of Oncology*. 2023;34:S755-S756.
  - ○ This pivotal trial led to the accelerated approval of this TROP2 ADC for previously treated EGFR-mutant non-small cell lung cancer
- Wang M, Fan Y, Sun M, et al. Sunvozertinib for patients in China with platinum-pretreated locally advanced or metastatic non-small-cell lung cancer and EGFR exon 20 insertion mutation (WU-KONG6): single-arm, open-label, multicentre, phase 2 trial. *Lancet Respir Med*. 2024;12(3):217-224. 10.1016/S2213-2600(23)00379-X
  - ○ This investigational trial led to the accelerated approval of this selective EGFR inhibitor, including against mutations of resistance

## Data Availability

No datasets were generated or analysed during the current study.
